# PDGF, NT-3 and IGF-2 in Combination Induced Transdifferentiation of Muscle-Derived Stem Cells into Schwann Cell-Like Cells

**DOI:** 10.1371/journal.pone.0073402

**Published:** 2014-01-14

**Authors:** Yi Tang, Hua He, Ning Cheng, Yanling Song, Weijin Ding, Yingfan Zhang, Wenhao Zhang, Jie Zhang, Heng Peng, Hua Jiang

**Affiliations:** 1 Department of Plastic Surgery, Changzheng Hospital, Second Military Medical University, Shanghai, China; 2 Department of Plastic Surgery, No. 411 Hospital of CPLA, Shanghai, China; 3 Department of Neurosurgery, Changzheng Hospital, Second Military Medical University, Shanghai, China; 4 Department of Transfusion, Changhai Hospital, Second Military Medical University, Shanghai, China; Department of Hematology, XinHua Hospital, Affiliated to Shanghai Jiao Tong University (SJTU) School of Medicine, Shanghai, China; 6 Department of Mathematics, Hong Kong Baptist University, Kowloon, Hong Kong; IBMC - Institute for Molecular and Cell Biology, Portugal

## Abstract

Muscle-derived stem cells (MDSCs) are multipotent stem cells with a remarkable long-term self-renewal and regeneration capacity. Here, we show that postnatal MDSCs could be transdifferentiated into Schwann cell-like cells upon the combined treatment of three neurotrophic factors (PDGF, NT-3 and IGF-2). The transdifferentiation of MDSCs was initially induced by Schwann cell (SC) conditioned medium. MDSCs adopted a spindle-like morphology similar to SCs after the transdifferentiation. Immunocytochemistry and immunoblot showed clearly that the SC markers S100, GFAP and p75 were expressed highly only after the transdifferentiation. Flow cytometry assay showed that the portion of S100 expressed cells was more than 60 percent and over one fourth of the transdifferentiated cells expressed all the three SC markers, indicating an efficient transdifferentiation. We then tested neurotrophic factors in the conditioned medium and found it was PDGF, NT-3 and IGF-2 in combination that conducted the transdifferentiation. Our findings demonstrate that it is possible to use specific neurotrophic factors to transdifferentiate MDSCs into Schwann cell-like cells, which might be therapeutically useful for clinical applications.

## Introduction

Schwann cells (SCs) play a crucial role in peripheral nerve development and regeneration, and are thus an attractive therapeutic target in peripheral nerve injuries [1]–[3]. It is reported that cultured SCs could induced neuronal sprouting and regrowth in cell culture experiments and improve peripheral nerve regeneration in vivo [4], [5]. SCs can be obtained from nerve biopsies for autologous transplantation and will not elicit an intense immune response. However, it's difficult to culture sufficient numbers of autologous SCs because of their restricted mitotic activity, and there are also other disadvantages such as limitations in the supply of nerve material [6], [7]. Use of allogeneic cells would need subsequent clinical immunosuppression [4]. Stem cells may be an alternative source for SCs. However, the clinical application of embryonic stem cells is limited because of ethical problems and their carcinogenic potential [8]. Increase evidence shows that adult stem cells may be promising candidate sources of cells [9], [10].

Skeletal muscle may represent a convenient and valuable source of stem cells for stem cell-mediated gene therapy. Previous evidence supports the existence of MDSCs that exhibits both multipotentiality and self-renewal capabilities and therefore can be used for tissue engineering and regenerative therapy [11], . MDSCs have the ability to differentiate, upon stimulation with defined media, into multiple types of cells, including myogenic, hematopoietic, osteogenic, adipogenic, and chondrogenic-like cells [13]. The apparent advantages of MDSCs have led us to investigate whether they could be transdifferentiated to a Schwann cell phenotype.

Our aim was to assess the phenotypic and bioassay characteristics of MDSCs transdifferentiated to SC-like cells. Importantly, we also sought to determine the neurotrophic factors which directed the transdifferentiation.

## Materials and Methods

### Ethics Statement

All animal experiments were approved by the Administrative Committee of Experimental Animal Care and Use of Second Military Medical University (SMMU, Licence No. 2011023), and conformed to the National Institute of Health guidelines on the ethical use of animals.

### Isolation and culture of mouse MDSCs

Primary muscle cultures were prepared from newborn (3–5 d) normal C57BL/6 mice, and the MDSCs were purified from the primary culture using a previously described preplate technique [11]. Skeletal muscle was dissected under a light microscopy followed by an enzymatic dissociation. The muscle cells were centrifuged, resuspended and cultured. Different populations of muscle-derived cells were isolated based on their adhesion characteristics. The muscle cells were plated on collagen-coated flasks for 24 h (pp1). The nonadherent cells were then transferred to other flasks (pp2). After 24 h, the floating cells in pp2 were collected, centrifuged, and plated on new flasks (pp3). These procedures were repeated at 24-h intervals until serial preplates (pp4–6) were obtained.

### Cell viability and growth assay of mouse MDSCs

Cell viability of mouse MDSCs were measured by trypan blue dye exclusion method. Trypan blue is a dye that cann't enter cells with an intact membrane and therefore stains only the cells with membrane disruption. The MDSCs were stained with 0.025% Trypan blue in PBS. The number of cells was counted using a hemocytometer.

Cell growth curves of mouse MDSCs (PP6 cells) were estimated by counting cell numbers every 24 hours. Cell numbers at individual time points were normalized to those at 0 day.

### Conditioned medium of mouse SCs

Primarily cultured mouse SCs were obtained as previously reported [6]. The sciatic nerve and dorsal root ganglia of six mice were isolated and dissected followed by an enzymatic dissociation. The cells were centrifuged, resuspended and cultured. Cytarabine (10 µM) was added in the medium to suppress fibroblast growth. The medium was changed each two days. After 3–4 days, half of the medium was collected and replaced with fresh medium. The conditioned medium of SCs was obtained by repeating the collection three times.

### Transdifferentiation of MDSCs to SC-like cells

Cultured MDSCs were stimulated with SC conditioned medium or medium containing different combinations of neurotrophic factors for three days. The morphological changes of the cells were studied using a light microscopy. Expression of SC markers S100, GFAP and p75 in the cells were analyzed by immunocytochemistry, flow cytometry and immunoblot.

### Immunocytochemistry

The primary antibodies used in this study were rabbit anti-desmin (1∶50, Cell Signaling), rat anti-Sca-1 (1∶40, Sigma-Aldrich), mouse anti-S100 (1∶40, Invitrogen). Cultured mouse MDSCs, SCs and tMDSCs were fixed and stained according to standard procedures [14].

### Flow cytometry

The percentages of Sca-1 and desmin positive MDSCs were analyzed by flow cytometry. All antibodies used in this assay were from eBioscience. Live cell events were collected and analyzed on a FACSCalibur flow cytometer using Cell Quest software.

S100, GFAP and p75 positive SCs or tMDSCs were analyzed using a similar protocol.

### Immunoblot

Total proteins were extracted from cells (Cultured MDSCs, SCs, and tMDSCs) using sodium dodecyl sulfate lysis buffer. The protein were electrophoresed on SDS/PAGE and transferred to polyvinyldifluoridine membranes. The membranes were incubated with the primary antibodies followed by the horseradish peroxidase–conjugated anti-rabbit or anti-mouse secondary antibodies. The protein bands were visualized using the ECL system and scanned.

### Enzyme-linked immunosorbent assay (ELISA)

The quantities of neurotrophic factors in the SC conditioned medium were measured using ELISA kit from Invitrogen. The tested medium was incubated in plates coated with capture antibodies (anti-BDNF, anti-PDGF, anti-NT-3, anti-IGF-2, anti-NGF, anti-GDNF, anti-FGF). After that, plates were incubated with secondary antibodies and then with peroxidase-conjugated anti-mouse IgG. Soluble colorimetric product was measured.

### Statistical analysis

Statistical differences between two groups were determined by two-tailed Student's *t* test. Multiple group comparisons were made by ANOVA test, using a significance level of 95%. Data were presented as means ± standard error of the mean.

## Results

### Isolation and Characterization of mouse MDSCs

**Figure 1:**
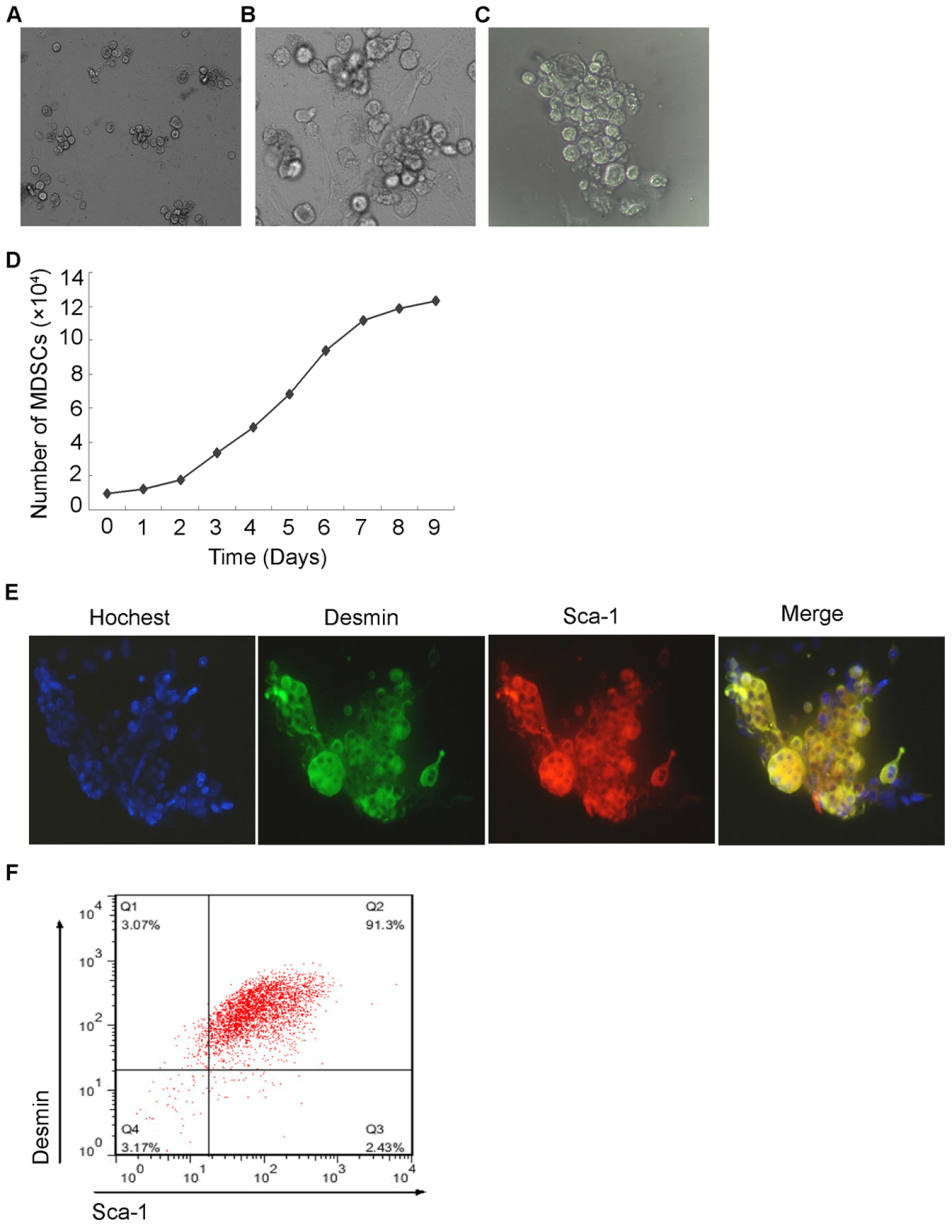
Isolation and Characterisation of mouse MDSCs. (A) Phase-contrast micrographs of undifferentiated PP6 cells isolated from mouse skeletal muscle. The cells had a rounded shape like stem cells. (B) Growth curve of PP6 cells showed a slow-cycling phenotype like stem cells. (C) Immunofluorescence staining showed the PP6 cells were desmin and Sca-1 positive which indicated they were MDSCs. (D) Flow cytometry assay demonstrated that 93.23±0.93% of the PP6 cells were Sca 1 positive, and 94.18±0.38% were desmin positive. 90.1±1.28% were double positive cells (data are mean % cells±S.E.M.). All experiments were repeated at least three times in triplicates.

The pre-plate technique was used to isolate different populations of muscle cells on the basis of their adherence to collagen-coated flasks [11], [15]. Consistent with the previous reports, the cells adhered early on (1^st^ preplate passage, PP1) were mostly fibroblasts without significant directional growth (Fig. S1). Myoblasts adhered at PP3 and satellite cells adhered at approximately PP5 (Fig. S1). The cells of PP6 had a more rounded shape like stem cells (Fig. 1 A). Cell viability determined by trypan blue staining demonstrated that over 95% of the PP6 cells were viable (Data not shown). Cell growth assay showed that the cells of PP6 had a quiescent slow-cycling phenotype which was also a feature of stem cells (Fig. 1 B). To further demonstrate that the PP6 cells were MDSCs, we investigated the expression of Sca1 which was a well-defined marker for putative MDSCs in PP6 cells. Immunocytochemistry showed that the PP6 are Sca 1 positive and are also desmin positive which indicated they were muscle-derived (Fig. 1 C). Flow cytometry assay demonstrated that 93.23±0.93% of the PP6 cells were Sca 1 positive, and 94.18±0.38% were desmin positive. 90.1±1.28% were double positive cells, indicating that most of the PP6 cells were MDSCs (Fig. 1 D). These results suggested that the PP6 cells isolated from mouse skeletal muscles were highly purified MDSCs.

### Transdifferentiation of mouse MDSCs to a Schwann cell phenotype

**Figure 2:**
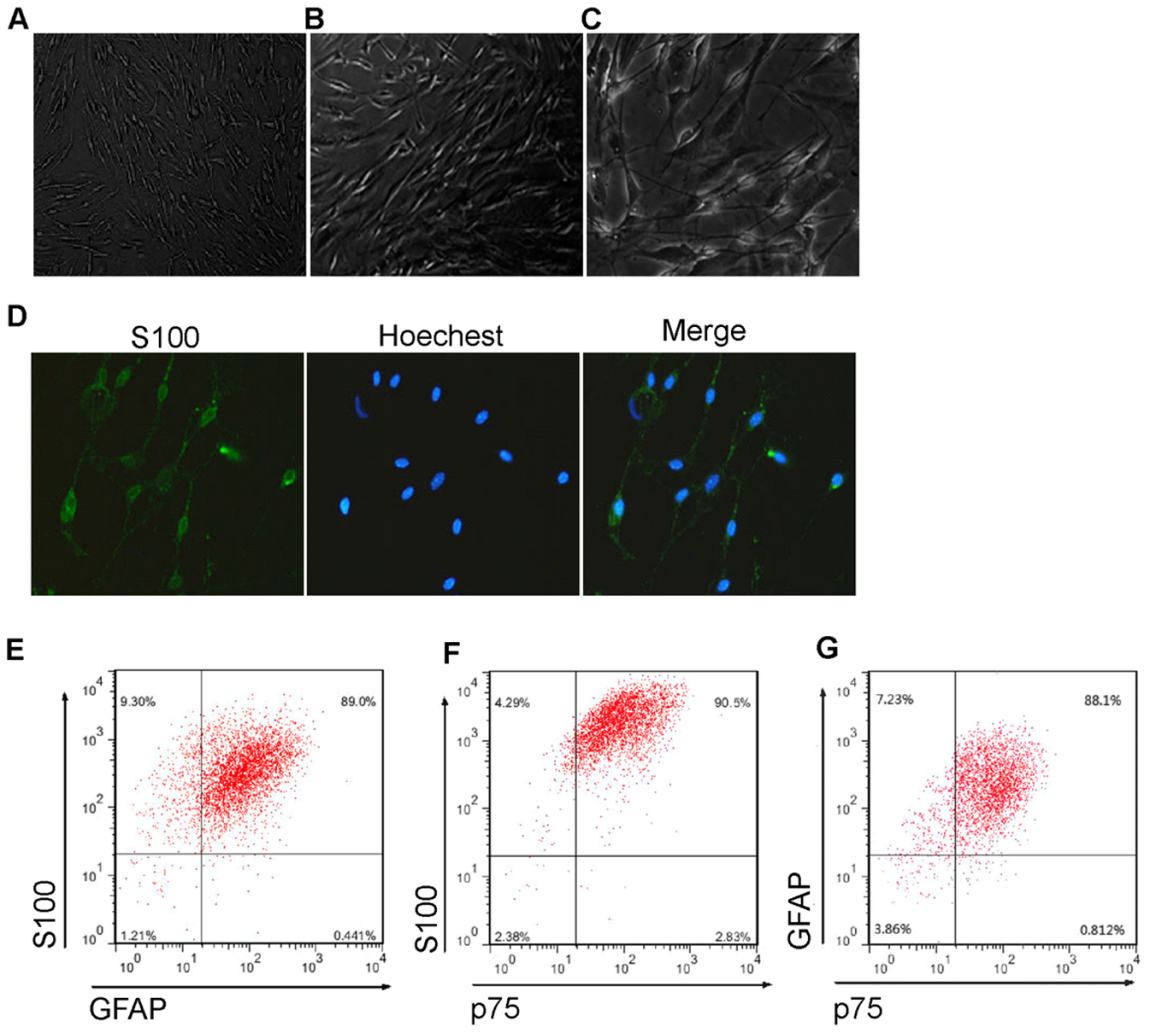
Isolation and Characterisation of mouse SCs to obtain the conditioned medium. (A) Phase-contrast micrographs of SCs isolated from mouse sciatic nerve and dorsal root ganglia. The cells had typical spindle-shaped SC morphology. (B) Immunofluorescence staining demonstrated the isolated cells expressed SC marker S100 protein. (C) Flow cytometry assay showed that most of the cells were S100 (96.77±1.46%), GFAP (92.92±4.94%), and p75 (93.38±0.90%) positive, and 86.12±1.53% of the cells expressed all of the three SC markers (data are mean % cells±S.E.M.).

To obtain conditioned medium of SC which would be used for differentiation of MDSCs, we isolated SCs from mouse sciatic nerve and dorsal root ganglia. The isolated cells had typical spindle-shaped SC morphology (Fig. 2 A). The isolated cells were assessed for expression of the SC markers S100, GFAP, and p75. Immunocytochemistry with anti-S100 demonstrated they highly expressed S100 (Fig. 2 B). In addition, flow cytometry assay showed that most of the cells were S100 (96.77±1.46%), GFAP (92.92±4.94%), and p75 (93.38±0.90%) positive, and 86.12±1.53% of the cells expressed all of the three proteins (Fig. 2 C). Thus, the isolated cells were SCs with high purity.

**Figure 3:**
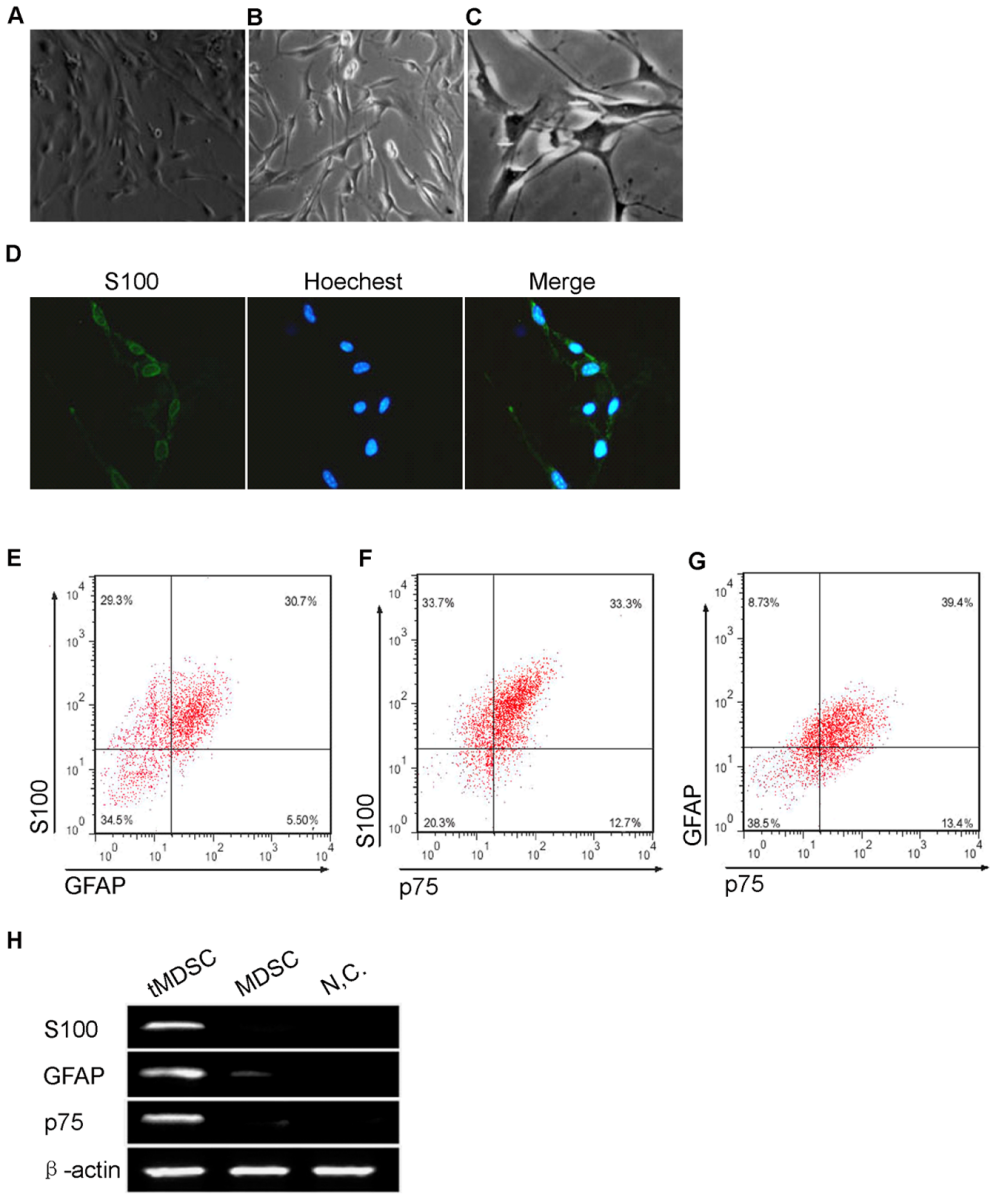
Transdifferentiation of mouse MDSCs to a Schwann cell phenotype. (A) Phase-contrast micrographs showed that MDSCs adopted a spindle-like shape with processes which was a characteristic of Schwann cells after treatment with SC conditioned medium. (B) Immunocytochemisty showed that the tMDSCs highly expressed the SC marker S100 protein. (C) Flow cytometry assay demonstrated that the portion of S100, GFAP, and p75 positive cells in tMDSCs were 65.48±6.20%, 39.84±1.66% and 41.08±0.78%, and 25.86±5.37% of the tMDSCs expressed all of the three SC markers (data are mean % cells±S.E.M.). (D) Immunoblot assay showed the MDSCs expressed of S100, GFAP and p75 only after transdifferentiation. NC, negative control. β-actin served as loading control. Right, bar graph of qualitative data in statistics. S100, GFAP and p75 protein levels were normalized to that of β-actin, shown as mean±S.E.M. **, p<0.01 vs. MDSC.

Next, the conditioned medium of isolated mouse SCs was added to the cultured MDSCs to induce their differentiation. 72 hours after the induction, morphology of MDSCs was changed to spindle-like shape with processes, which was a typical morphology of SC-like cells (Fig. 3 A). The transdifferentiated SC-like cells (tMDSCs) were assessed for expression of the SC markers S100, GFAP, and p75 to study evidence of phenotypic progression to a SC lineage. Immunocytochemisty showed that the tMDSCs highly expressed the SC marker S100 protein (Fig. 3 B). Moreover, flow cytometry assay demonstrated that the portion of S100, GFAP, and p75 positive cells were 65.48±6.20%, 39.84±1.66% and 41.08±0.78%, while 25.86±5.37% of the cells expressed all of the three proteins (Fig. 3 C). Immunoblot assay of the S100, GFAP and p75 expression showed an accord with flow cytometry (Fig. 3 D). The MDSCs expressed the SC markers highly only after transdifferentiation, indicating the tMDSCs were progressed along a SC lineage.

### Neurotrophic factors essential for the transdifferentiation

It has been reported that neurotrophic factors secreted by SCs support the survival of neurons cultured in vitro and in vivo after peripheral nerve injury [16], [17]. In this study, the conditioned medium of isolated mouse SC could induce the transdifferentiation of MDSCs to SC-like cells. We sought to determine the neurotrophic factors involved in this process.

**Figure 4:**
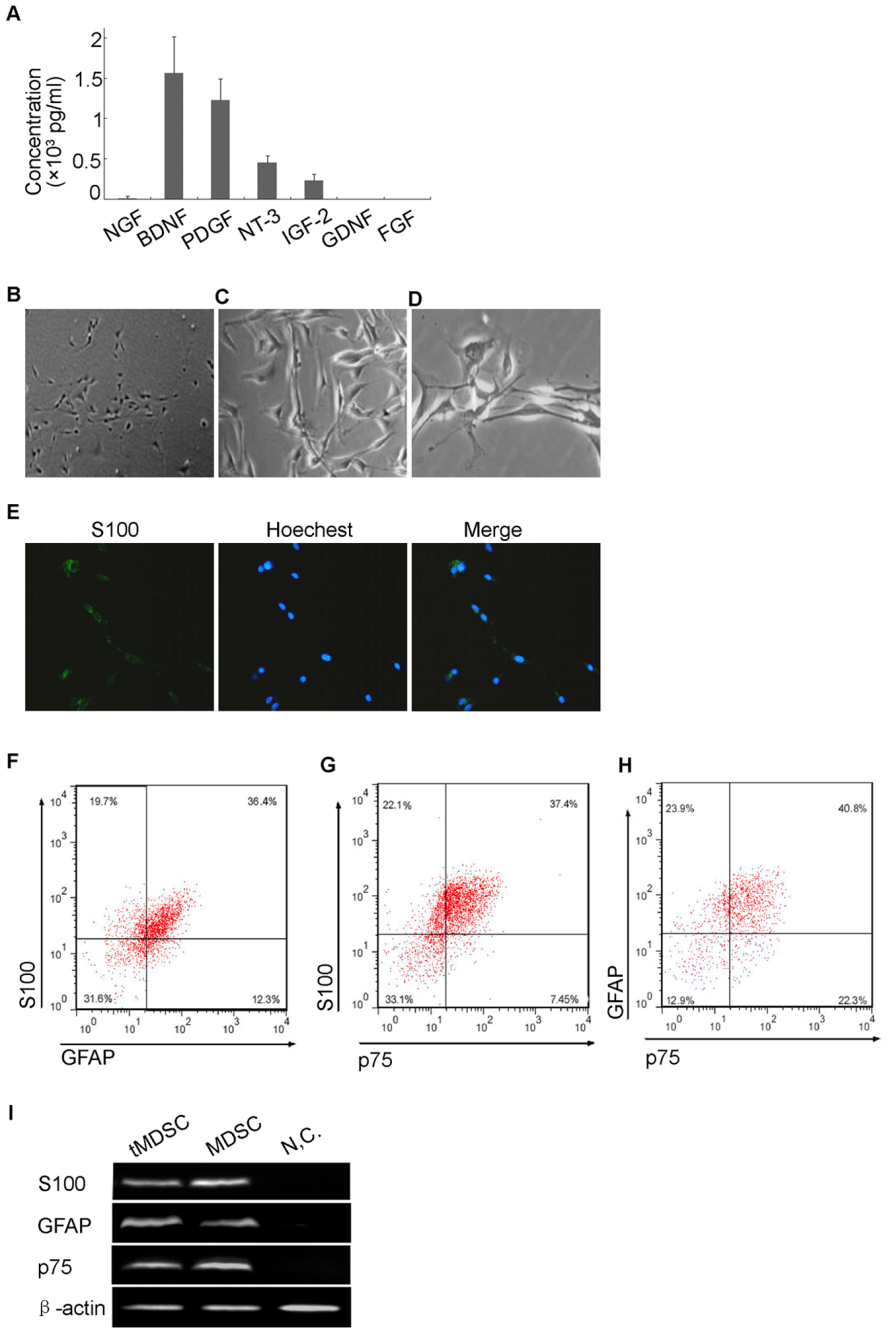
Neurotrophic factors which direct the transdifferentiation. (A) ELISA assay showed the quantity of seven neurotrophic factors in the SC conditioned medium. There were high levels of BDNF, PDGF, NT-3 and IGF-2. The levels of NGF were relatively low, while GDNF and FGF were undetectable. (B) Phase-contrast micrographs showed that MDSCs adopted a SC-like shape upon treatment with PDGF (1000 pg/ml), NT-3 (500 pg/ml) and IGF-2 (200 pg/ml) in combination. (C) Immunocytochemisty showed that the tMDSCs highly expressed the SC marker S100 protein. (D) Flow cytometry assay demonstrated that the portion of S100, GFAP, and p75 positive cells in tMDSCs were 58.64±4.38%, 47.38±0.84% and 44.33±2.39%, while 27.89±5.98% of the tMDSCs expressed all of the three SC markers (data are mean % cells±S.E.M.). (E) Immunoblot assay showed the MDSCs expressed of S100, GFAP and p75 only after transdifferentiation. β-actin served as loading control. Right, bar graph of qualitative data in statistics. S100, GFAP and p75 protein levels were normalized to that of β-actin, shown as mean±S.E.M. **, p<0.01 vs. MDSC.

First, we detected the quantity of seven neurotrophic factors in the conditioned medium, including nerve growth factor (NGF), brain-derived neurotrophic factor (BDNF), neurotrophin-3 (NT-3), glial cell line-derived neurotrophic factor (GDNF), platelet-derived growth factor (PDGF), fibroblast growth factor (FGF) and insulin-like growth factor-2 (IGF-2). Quantitative method of ELISA was conducted and the results showed that there were high levels of BDNF, PDGF, NT-3 and IGF-2 in the conditioned medium (Fig. 4 A). The levels of NGF were relatively low, while GDNF and FGF were undetectable (Fig. 4 A). It seemed that BDNF, PDGF, NT-3, IGF-2 and NGF might be involved in the transdifferentiation of MDSCs to SC-like cells.

To determine which neurotrophic factors directed the transdifferentiation, we treated the MDSCs with one alone, two in combination or three in combination of the five neurotrophic factors and detect the morphological changes of the cells. None of the five neurotrophic factors could induce morphological changes of MDSCs alone, and the results were similar when MDSCs were treated with two factors in combination (data not shown). In the treatments of three neurotrophic factors in combination, only the combination of PDGF (1000 pg/ml), NT-3 (500 pg/ml) and IGF-2 (200 pg/ml) could induce the morphological change of MDSCs to SC-like cells (Fig. 4 B). Moreover, immunocytochemistry showed that PDGF, NT-3 and IGF-2 in combination also induced expression of SC marker S100 protein in MDSCs (Fig. 4 C). The results of flow cytometry were consistent with the SC conditioned medium induced transdifferentiation (Fig. 3 C), as the portion of S100, GFAP, and p75 positive cells were 58.64±4.38%, 47.38±0.84% and 44.33±2.39%, while 27.89±5.98% of the cells expressed all of the three proteins (Fig. 4 D). In addition, immunoblot assay demonstrated that treatment of PDGF, NT-3 and IGF-2 in combination could induced high expression of S100, GFAP, and p75 in transdifferentiated MDSCs (tMDSCs) (Fig. 4 E). These results indicated the effects of SC conditioned medium on MDSCs transdifferentiation might be mediated by PDGF, NT-3 and IGF-2 in combination.

## Discussion

In this study, we showed that primarily cultured mouse MDSCs could be transdifferentiated to Schwann cell phenotype. The transdifferentiation could be induced by Schwann cell conditioned medium or by the combined treatment of three neurotrophic factors. Several kinds of evidence support the success of transdifferentiation. First, we observed that morphology of MDSCs was changed along a typical SC-like spindle-like shape with processes after the transdifferentiation. Second, we showed that the SC marker S100 was highly expressed in the tMDSCs using immunocytochemistry assay. Third, the immunoblot demonstrated clearly that the SC markers S100, GFAP and p75 were expressed highly only after the transdifferentiation. Finally, flow cytometry assay showed that the portion of S100 expressed cells was about 60 percent and over one fourth of the transdifferented cells expressed all the three SC markers, indicating an efficient transdifferentiation.

MDSCs are a potentially new type of undifferentiated cell isolated from skeletal muscle without myogenic restrictions. MDSCs have been reported differentiate into different types of cells, including myogenic, hematopoietic, osteogenic, adipogenic, and chondrogenic-like cells [13]. MDSCs also have a remarkable long-term self-renewal and regeneration capacity [18]. Skeletal muscle is also a convenient source which could not induce the problem of clinically immunosupression. These properties form the basis of potential clinical use of MDSCs in the therapies of degenerative diseases. In this study, we have successfully induced the transdifferentiation of MDSCs towards SC like phenotype, however, the true function of these SC like cells remains to be fully investigated.

Previous studies have shown that growth factors affect differentiation directly in stem cell populations [19]. However, there are not many reports about single factor which directs differentiation exclusively to one cell type. SCs developed through stages known as SC precursor cells, early SC and mature myelinating or non-myelinating SC. Several growth factors, such as bFGF, PDGF, neuregulin-1 (NRG-1) and its isoforms, neurotrophin-3 and IGF-1, have been reported involved in the development from SC precursor cells into early SC [20], [21]. Adipose-derived stem cells treated with a mixture of glial growth factors (GGF-2, bFGF, PDGF and forskolin) adopted a phenotype similar to Schwann cells [22]. Combinations of bFGF, PDGF-AA and Her-β have proved to successfully induce the differentiation of bone marrow stromal cells to SC like cells [23]. In this study, the combinations of PDGF, NT-3 and IGF-2 successfully induced transdifferentiation of MDSCs along SC like phenotype. From the neurotrophic factors detected in the Schwann cell conditioned medium, only this combination could induce the transdifferentiation. We also used the reported growth factors (GGF-2, bFGF, PDGF, forskolin, PDGF-AA and Her-β) for the transdifferentiation, but none of these growth factors or the reported combination had positive effects on MDSCs transdifferentiation. These results suggested that specific growth factors conduct the differentiation of different types of stem cells, even differentiation towards the same cell types.

## Supporting Information

Figure S1 **Morphology of cells in preplate method.**
(TIF)
