# Supplementary figures and images for: PDGF, NT-3 and IGF-2 in Combination Induced Transdifferentiation of Muscle-Derived Stem Cells into Schwann Cell-Like Cells
Source: PLoS One. 2014 Jan 14;9(1):e73402. doi: 10.1371/journal.pone.0073402 (PMC3891637; doi:10.1371/journal.pone.0073402)

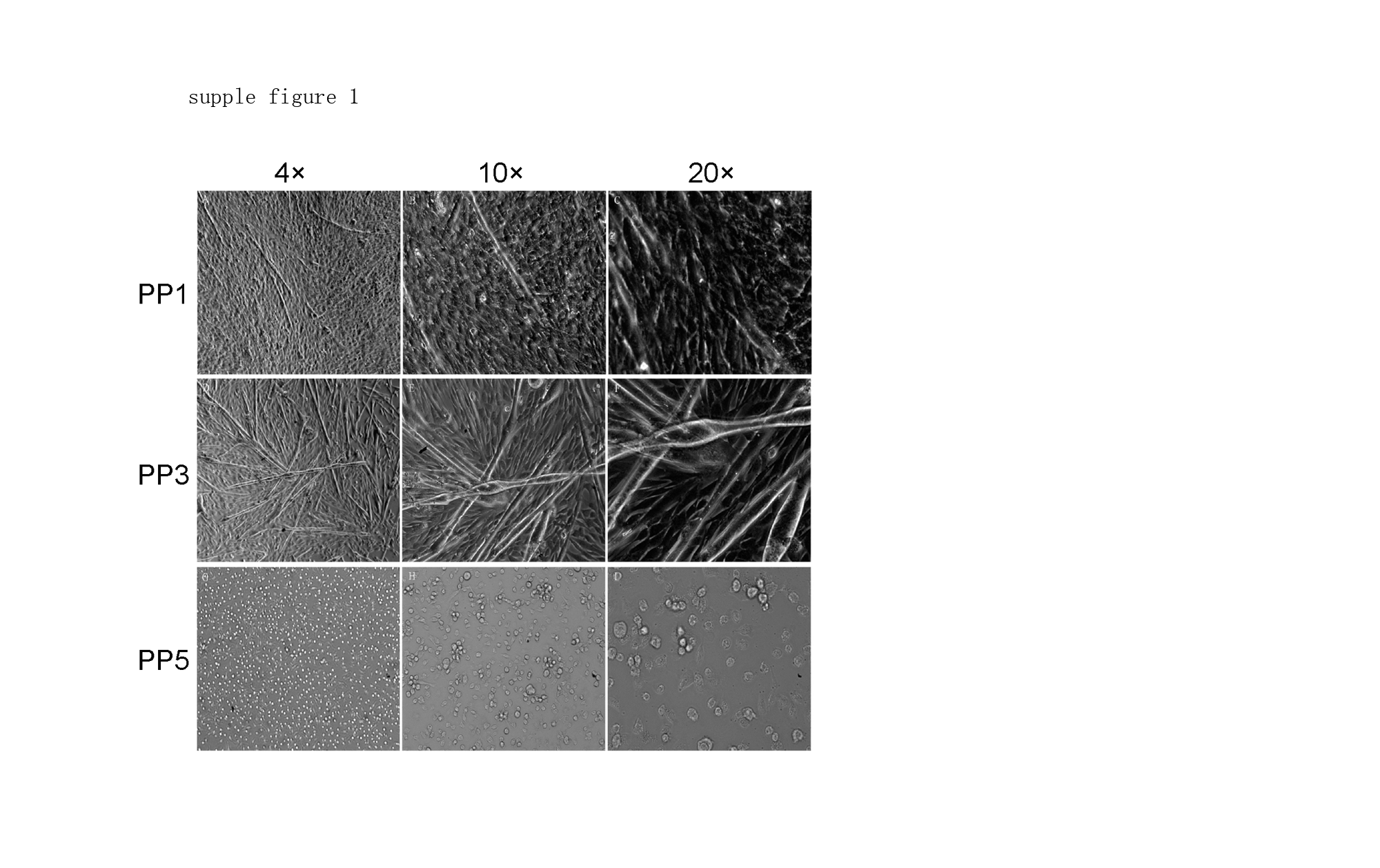

Supplement: Figure S1 — Morphology of cells in preplate method. (TIF) [file pone.0073402.s001.tif]
